# Construction of a Prognostic Model for Hypoxia-Related LncRNAs and Prediction of the Immune Landscape in the Digestive System Pan-Cancer

**DOI:** 10.3389/fonc.2022.812786

**Published:** 2022-04-27

**Authors:** Zikang He, Hongfeng Liu, Huilin Guan, Jinli Ji, Ying Jiang, Naiwen Zhang, Zheyao Song, Xingyun Wang, Ping Shen, Huan Wang, Rongjun Cui

**Affiliations:** ^1^Department of Biochemistry and Molecular Biology, Mudanjiang Medical University, Mudanjiang, China; ^2^Department of Scientific Research, Mudanjiang Medical University, Mudanjiang, China

**Keywords:** hypoxia, lncRNA, digestive system pan-cancer, prognostic model, immune microenvironment, bioinformatics

## Abstract

Digestive system pan-cancer is a general term for digestive system tumors including colorectal carcinoma (CRC), esophageal carcinoma (ESCA), stomach adenocarcinoma (STAD), and liver hepatocellular carcinoma (LIHC). Since the anatomical location, function and metabolism are closely related, there may be similarities in development and progression of these tumors. Hypoxia is the consequence of an imbalance between oxygen demand and supply, and intracellular hypoxia is associated with malignant progression, treatment resistance, and poor prognosis in tumors. Therefore, an urgent and challenging task is to investigate the molecular mechanisms associated with hypoxia in digestive system pan-cancer for the prognosis and treatment of digestive tract tumors. In this study, we identified 18 hypoxia-related lncRNAs (HRlncRNAs) by co-expression analysis between hypoxia genes and lncRNAs from digestive system pan-cancer. Six HRlncRNAs were then obtained using lasso regression and multivariate cox analysis to construct a prognostic model. Next, the Akaike information criterion (AIC) values for 3-year receiver operating curve (ROC) were counted to determine the cut-off point and establish an optimal model to distinguish between high- or low-risk groups among patients with digestive system pan-cancer. To evaluate the stability of the prognosis model, we validated it in terms of survival outcomes, clinicopathological stage, tumor-infiltrating immune cells, immune checkpoint inhibitors (ICIs) and anticancer drugs sensitivity. The results suggested that high- risk group had a worse prognosis and a more positive association with tumor-infiltrating immune cells such as B cells, cancer-associated fibroblasts, endothelial cells, monocytes, macrophages and bone marrow dendritic cells in digestive system pan-cancer. Immune checkpoint inhibitors (ICIs) related biomarkers discovered that high-risk group was positively correlated with high expression of HAVCR2 in digestive system pan-cancer. The anticancer drugs sensitivity analysis showed that the high-risk group was associated with the lower half-inhibitory centration (IC50) of Imatinib in digestive system pan-cancer. In conclusion, the prognostic model of HRlncRNAs showed a promising clinical prediction value and may provide a useful reference for the diagnosis and treatment of the digestive system tumors.

## Introduction

Digestive system pan-cancer is a general term for malignant tumors of the digestive system, which mainly includes esophageal carcinoma (ESCA), stomach adenocarcinoma (STAD), liver hepatocellular carcinoma (LIHC), colorectal carcinoma (CRC). Malignant tumors of the digestive system are by far the most common cause of cancer death (1), and are characterized by the lack of obvious early symptoms, difficulty in clinical diagnosis, and extremely low survival rates at late stages. According to 2018 statistics, malignant tumors of the digestive system accounted for more than a quarter (26.3%) of the global cancer incidence and more than a third (35.4%) of all cancer-related death (2). However, clinical treatment of digestive system malignancies has yielded some achievements, particular with immune checkpoint inhibitors (ICIs) therapies. For instance, the binding of programmed death 1 (PD-1) to programmed death ligand 1 (PD-L1) suppresses the immune microenvironment surrounding tumor tissue, leading to a downregulation of specific T-cell activity and contributing to immune escape. Nivolumab and Pembrolizumab, as PD-1 and PD-L1 inhibitors, can block the pathway and activate the anti-tumor immune response, thus curbing tumor growth (3).

Hypoxia, and in particular hypoxia-inducible factor 1 alpha (HIF-1α) is a common feature of solid tumors and is involved in metabolic reprogramming of tumors, stem cell characteristics, angiogenesis, extracellular matrix organization and metastasis of cancers (4–7). Moreover, because of abnormal proliferation of tumor cells resulting in increased oxygen consumption, the hypoxic environment promotes the generation of resistance to cancer therapy, which is strongly related to poor clinical prognosis (8, 9). Hypoxia is known to be inextricably linked to the immune microenvironment (TME), which affects the effectiveness of immunotherapy through a variety of mechanisms, including innate and adaptive immunity. Hypoxia contributes to the evasion of innate immunity by increasing the expression of the metalloproteinase ADAM10 in tumor cells in dependence on the accumulation of HIF-1α (10). In adaptive immunity, hypoxia leads to reduced differentiation of CD4+ T effector cells and enhanced the suppressive capacity of regulatory T cells (11). Therefore, a critical element for the benefit of tumor immunotherapy is the amelioration of hypoxic conditions.

Long non-coding RNAs (lncRNAs) are RNA molecules with transcripts greater than 200 nucleotides, which are not directly involved in gene coding and protein synthesis. However, they can regulate gene expression at the epigenetic, transcriptional, and post-transcriptional levels, and thus modulate tumor cell promoting or suppressing effects (12). Previous studies have shown that lncRNAs, which also can induce hypoxic signaling, facilitate the adaptation of tumor cells to the hypoxic environment. For instance, Zhang et al. found that lncRNA NEAT1 promotes hepatocellular carcinoma growth by regulating miR-199a-3p/UCK2 under hypoxic conditions (13). Zhu et al. suggested that hypoxia-induced cisplatin resistance can be controlled by regulating lncRNA EMS/miR-758-3p/WTAP axis in esophageal cancer (14). LncRNA CPS1-IT1 inhibits epithelial mesenchymal transition (EMT) and metastasis of colorectal cancer by inactivating HIF-1α and suppressing hypoxia-induced autophagy (15). LncRNA PCGEM, serves as a hypoxia-induced factor, promotes the proliferation and migration of gastric cancer by regulating SNAI1 (16). Recent researches focused on hypoxia-related lncRNAs (HRlncRNAs) promoting tumor growth and metastasis by enhancing certain immunogenic traits of TME and driving immune escapes (17, 18). No research has yet been conducted to analyze the application values of HRlncRNAs for clinical immunotherapy from the viewpoint of the digestive system pan-cancer.

In this study, the prognostic model was constructed using six HRlncRNAs from pan-cancer of the digestive system by machine learning. Moreover, we assessed their prognostic value in terms of survival outcomes, clinical characteristics, immune tumor invasion and anticancer drugs sensitivity, providing a new perspective for the diagnosis and treatment of digestive system tumors.

## Material and Methods

### Data Download and Extraction

We downloaded the transcriptome profiling data of four digestive system carcinomas from The Cancer Genome Atlas (TCGA) database (https://portal.gdc.cancer.gov/), including 11 normal and 160 tumor of samples in ESCA, 32 normal and 375 tumor of samples from STAD, 50 normal and 374 tumors of samples from LIHC, 51normal and 641 tumor of samples from CRC. LncRNAs from each of the four tumors were extracted using the Perl and R languages. In addition, we downloaded and combined the complete clinical information of patient with four digestive tumors from the TCGA database. In screening of clinical information, we excluded samples with a follow-up time of less than 50 days. Finally, 1368 clinical samples of digestive pan-cancer were included for subsequence analysis.

In the validation phase of clinical outcomes, we randomly divided the 1368 clinical data into training and validation sets using “caret” package in the R languages, and performed reproducibility analysis, respectively.

As the data involved in this research were obtained from TCGA database and strictly follow TCGA publication guidelines (http://cancergenome.nih.gov/abouttcga/policies/publicationguidelines), the approval of the ethics committee was not required.

### Screening of Co-Expression HRlncRNAs

The hypoxia gene sets were downloaded from Gene Set Enrichment Analysis website (19) (GSEA, http://www.gsea-msigdb.org/gsea/index.jsp). Pearson’s correlation coefficient was used to calculate the correlation between lncRNAs and hypoxia genes in the four digestive system carcinomas, respectively. The square of correlation coefficient |R^2^| > 0.4 and P < 0.001 was considered being HRlncRNAs. Next, co-expressed HRlncRNAs were obtained by intersection of lncRNAs of those using Draw Venn Diagram (http://bioinformatics.psb.ugent.be/webtools/Venn/). Moreover, we combined and normalized the expression values of co-expressed HRlncRNA samples from the four digestive malignancies for the subsequent comprehensive analysis of digestive pan-cancer.

### Establishment of a Prognostic Model to Evaluate the Risk Score

The univariate cox analysis was first performed on the co-expressed HRlncRNAs. Subsequently, HRlncRNAs with P < 0.05 were included into least absolute shrinkage and selection operator (Lasso) regression and multivariate cox analysis, as well as for construction of the model. The AUC values of the model were also calculated and were drawn as 1-, 2-, and 3-year ROC curves. When the curve reached the highest point, indicating the maximum AUC value, the calculation procedure was terminated while the model was taken as the optimal candidate. We calculated the risk score of all clinical cases using the constructed risk model and the calculation formula was as follows: Risk Score= $\sum_{i=1}^{n}\beta i*\left(expression\;of\;HRlncRNAi\right)$ . In which, *β* stand for the correlation coefficient of HRlncRNA. The AIC value of the 3-year ROC curve were assessed to determine the maximum inflection point, which was considered as the cut-off point (0.935) to distinguish between the high- or low-risk groups.

### Validation of the Constructed Prognostic Model

To validate whether the cut-off point was plausible, we performed Kaplan-Meier analysis to illustrate the survival difference in the high- or low-risk groups, and plotted the survival curve. Also, the chi-square test was performed to analyze the relationship between the prognostic model and the clinicopathological characteristics. Wilcoxon signed-rank test was used to calculate the risk score differences among different these clinicopathological characteristics. Univariate and multivariate cox regression analyses were then conducted to assess whether the prognostic model could be used as an independent predictor of clinical prognosis.

### Investigation of the Relationship Between Tumor-Infiltrating Immune Cells and the Prognostic Model

To analyze the relationship between the risk model and tumor-infiltrating immune cells, we calculated the immune-cells infiltration of samples from the digestive pan-cancer dataset using seven methods, including XCELL, TIMER, QuanTIseq, MCPcounter, EPIC, CIBERSORT-ABS and CIBERSORT. The Wilcoxon signed-rank test was used to analyze the difference in the content of immune infiltrating cells between the high- and low-risk groups of the constructed model. Spearman correlation analysis was performed to analyze the relationship between the risk scores and the tumor-infiltrated immune cells. The threshold of significance was set at p <0.05.

### Analyses of the Immunosuppressive Molecules Expressing Related to ICIs

To investigate the relationship between the risk model and the expression level of ICIs-related genes, such as HAVCR2, CD274, PDCD1, CTLA4, TIGIT, and LAG3. We utilized the “ggstatsplot” package of R software and violin plots for visualization.

### Exploration of the Significance of the Prognostic Model in the Clinical Treatments

To evaluate the efficacy of the risk model in the clinical treatments for digestive system pan-cancer, we analyzed the correlation between prognostic models and the IC50 of commonly administered anticancer drugs for the ESCA, STAD, LIHC and CRC datasets in the TCGA project, respectively. The anti-tumor drugs such as Imatinib, Doxorubicin, Mitomycin, Gemcitabine and Cisplatin are recommended for digestive system cancers treatment by NCCN guidelines. The difference in the IC50 between the high- and low-risk groups was compared by Wilcoxon signed-rank test and the results are shown as box drawings obtained using “pRRophetic” and “ggplot2” packages of R software.

## Results

### Identification of Co-Expressed HRlncRNAs of Four Digestive System Carcinomas

First, the RNA transcriptome data were downloaded from TCGA database, including ESCA, STAD, LIHC and CRC. Next, co-expression analysis was performed between known 243 hypoxia genes and lncRNAs of four digestive system tumors. 1133 HRlncRNAs were identified in ESCA. For STAD, 866 HRlncRNAs were identified. 850 HRlncRNAs were identified in LIHC. For CRC, 927 HRlncRNAs were identified. It intersected them in four digestive system cancers, resulting in 18 HRlncRNAs overlapping (|R^2^| > 0.4, P < 0.001).

### Construction of HRlncRNAs Prognostic Model

**Figure 1:**
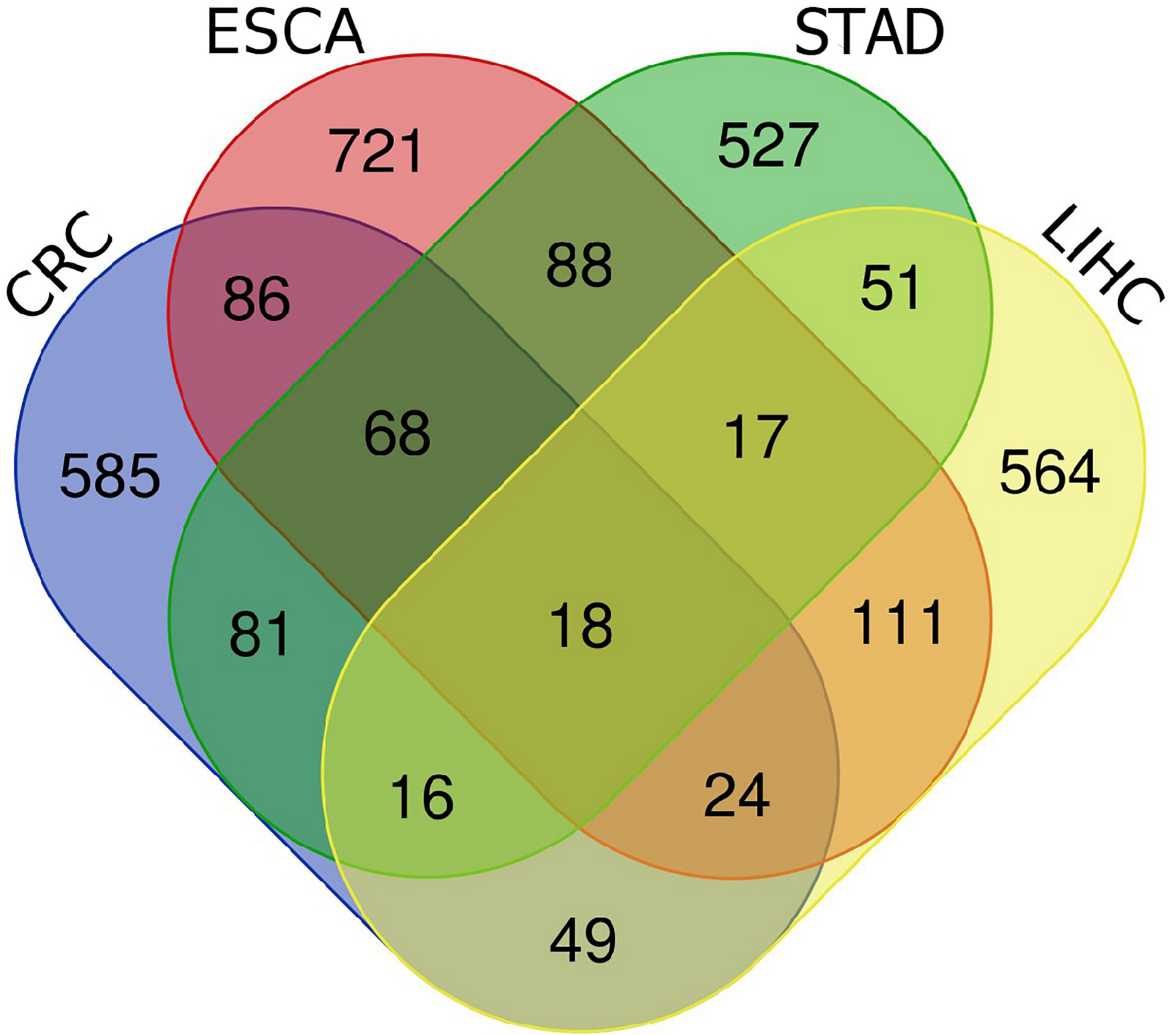
The venn diagrams of four digestive system cancers. A total of 18 HRlncRNAs were obtained from CRC, ESCA, STAD and LIHC.

**Figure 2:**
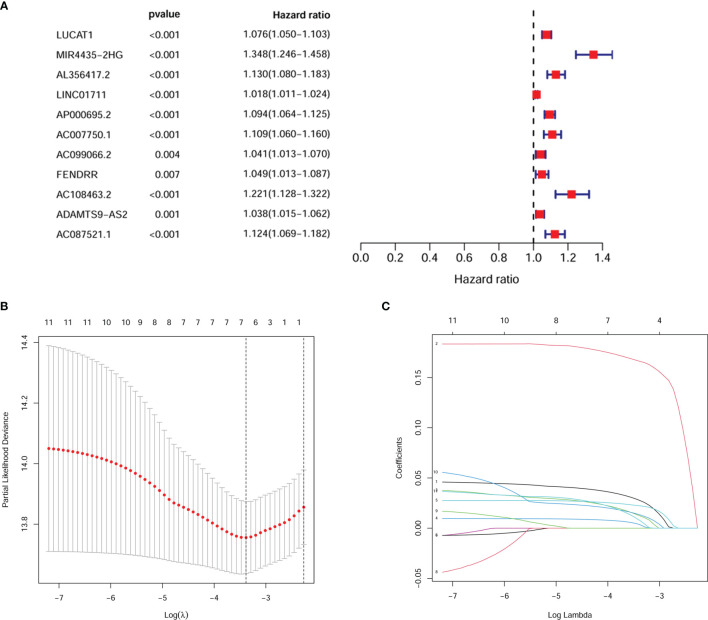
Screening for pivotal HRlncRNAs. **(A)** 11 HRlncRNAs were derived by univariate cox analysis. **(B, C)** It predicted the optimal model using Lasso regression analysis.

We first combined the expression of 18 HRlncRNAs normalized to four digestive cancers (**Figure 1**). According to the result of univariate cox analysis, 11 HRlncRNAs had significantly prognostic value for the patients with digestive system pan-cancer (P < 0.05, **Figure 2A**). Subsequently, six HRlncRNAs were included in the prognostic model by the Lasso regression and multivariate cox analysis (**Figure 2B** and **Table 1**). The expression of HRlncRNAs weighted by the multivariate cox regression coefficient were transformed into a risk score as follows: risk score = (0.045*expression of LUCAT1) + (0.194*expression of MIR4435-2HG) + (0.010*expression of LINC01711) + (0.033*expression of AP000695.2) + (0.030*expression of ADAMTS9-AS2) + (0.049*expression of AC087521.1). 

**Table 1:** the correlation coeffcients for six HRlncRNAs gaind by multivariate cox analysis.

lncRNA	coef	HR	HR.95L	HR.95H	pvalue
LUCAT1	0.045	1.046	1.013	1.080	<0.01
MIR4435-2HG	0.194	1.214	1.094	1.347	<0.01
LINC01711	0.010	1.010	1.002	1.017	<0.01
AP000695.2	0.033	1.034	0.991	1.078	0.12
ADAMTS9-AS2	0.030	1.031	1.004	1.058	0.02
AC087521.1	0.049	1.050	0.988	1.116	0.12

**Figure 3:**
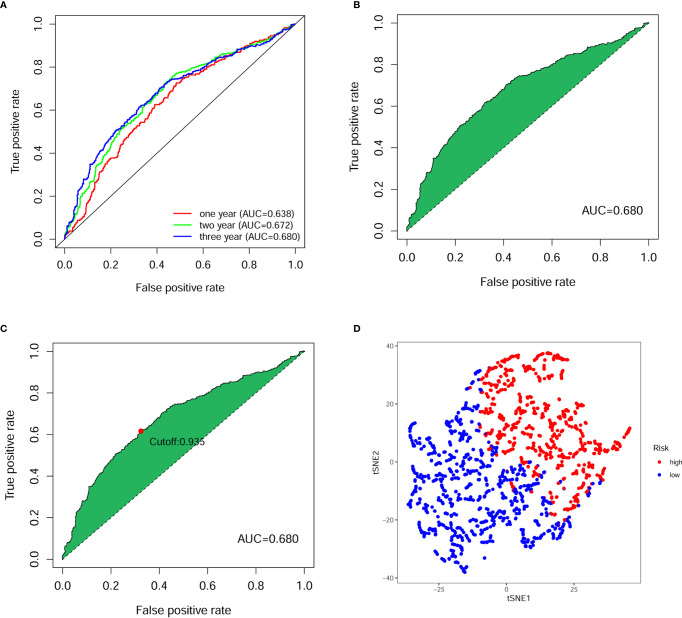
Evaluation of prognostic model for digestive system pan-cancer. **(A)** The ROC analysis showed that the AUCs of 1-, 2- and 3-year survival of patients with digestive system cancer were greater than 0.6. **(B)** The AUC for ROC analysis of the optimal model was 0.680. **(C)** The cut-off point calculated using the AIC was the maximum inflection point of the optimal model. **(D)** The tSNE down-scaling analysis showed strong heterogeneity between high- and low-risk groups distinguished using truncation points.

Next, we calculated the area under the curve (AUC) of the receiver operating characteristic curve (ROC) for the risk score-based prognostic model, plotted the curve and found the highest point to be 0.680 (**Figures 3A, B**). To verify the optimality of the model, we drew ROC curves for 1-, 2- and 3-years related to survival, which revealed that all AUC areas were above 0.6 (**Figure 3C**). The largest inflection point of the 3-year ROC curve was then identified as the cut-off value using the Akaike information criterion (AIC) value. Based on the cut-off point (0.935), the samples of patients were divided into two risk groups, comprising 582 high-risk and 786 low-risk groups, respectively. The tSNE downscaling analysis demonstrates superior heterogeneity in high- and low-risk score groups, indicating that the efficacy of the cut-off point that distinguish the between high- and low-risk groups (**Figure 3D**).

### Prediction of the Robustness of HRlncRNAs Prognostic Model

**Figure 4:**
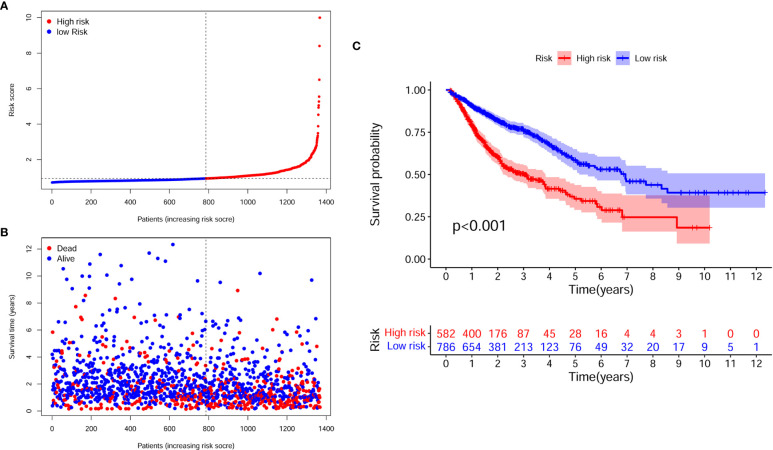
The assessment of prognostic model for survival outcomes. **(A)** As the number of patients increased, the risk scores of the prognostic model were raised. **(B)** As the number of patients increased, the number of patient deaths significantly increased. **(C)** The survival time was significantly longer in the low-risk group than in the high-risk group.

**Figures 4A, B** illustrate the distribution of high- and low-risk scores among the 1368 cases. We found that as the risk score increased, the mortality rate of the patients gradually increased. Kaplan-Meier analysis showed that the risk score significantly corrected with overall survival (OS) in patients with digestive system pan-cancer. The low-risk score group had a better prognostic outcome than the high-risk group (P < 0.001) (**Figure 4C**).

Next, we utilized multivariate cox analysis to construct prognostic models and calculated risk scores for the training and validation sets, respectively. The two datasets were classified into high- and low-risk groups based on the pre-existing cut-off point (0.935), separately. It was found that the training and validation sets were consistent with the above results, which revealed that the prognostic model for the digestive pan-cancer had a stronger predictive value (**Figure S1**).

### Assessment of Clinicopathological Characteristics of Prognostic Model

**Figure 5:**
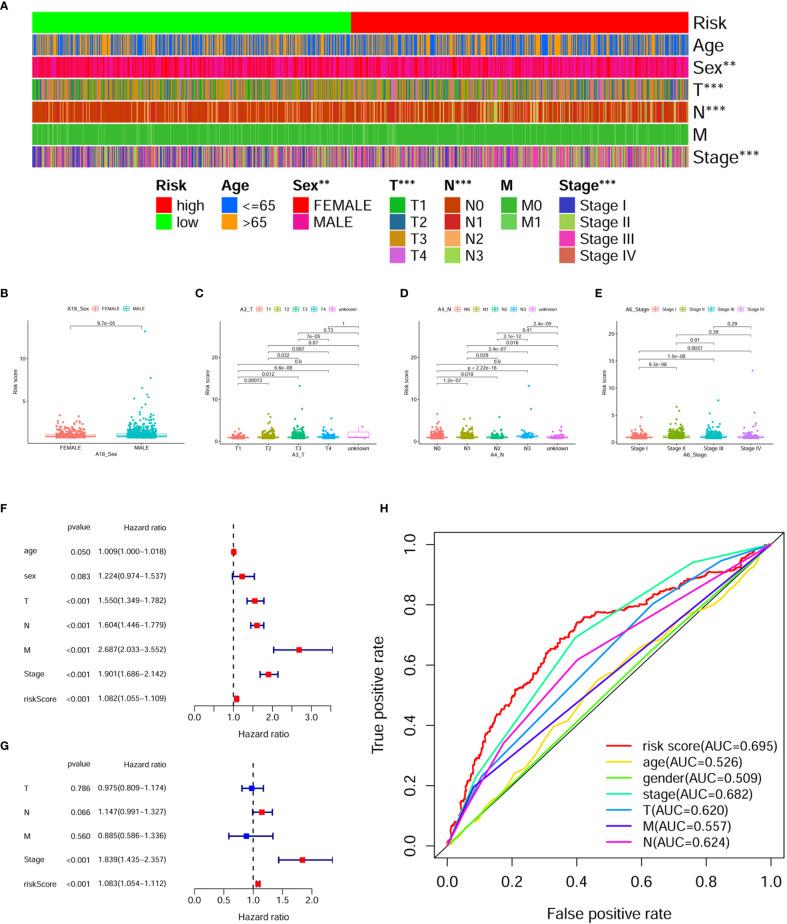
Assessing the relationship between clinicopathological characteristics and prognostic models. **(A)** The bar chart illustrates it remarkably correlated the risk score with sex, T stage, N stage, and clinical stage. **(B–E)** The scatterplot shows that **(B)** sex, **(C)** T stage, **(D)** N stage and **(E)** clinical stage are significantly associated with risk score. **(F)** The risk score, clinical stage, T stage, N stage, and M stage were significantly associated with clinical prognosis in digestive system pan-cancer by the univariate cox analysis. **(G)** The risk score and clinical stage as independent prognostic factors for digestive system pan-cancer according to the multivariate cox analysis. **(H)** The ROC analysis based on the risk score and clinicopathological features demonstrated that the AUCs for both risk scores (0.695) and clinical staging (0.682) were greater than 0.65.

**Figure 6:**
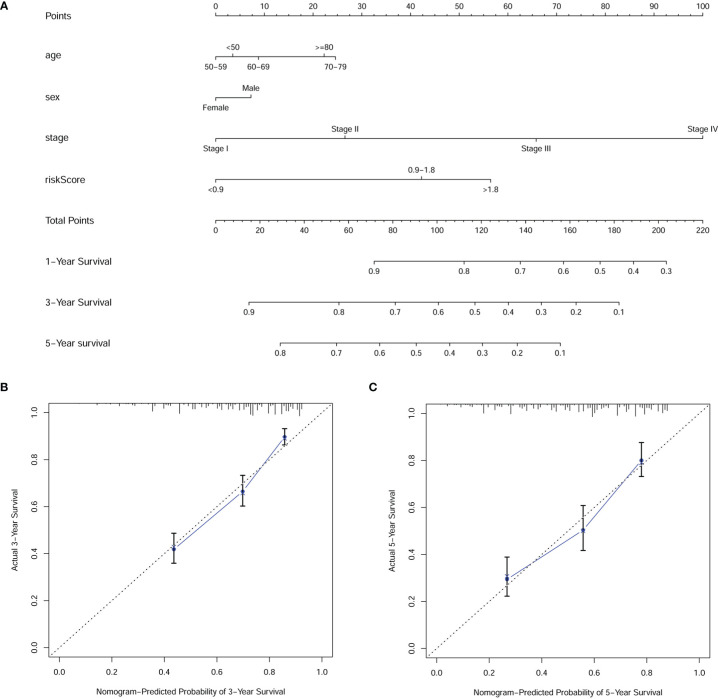
The **(A)** nomogram and **(B, C)** calibration plots of 3 and 5 years demonstrated that the combination of risk score and clinicopathological characteristics could well predict the prognostic performance of patients with digestive system pan-cancer.

To better validate the robustness of the prognostic model, we investigated the relationship between risk score and clinicopathological characteristics using chi-square tests. The bar chart shows that sex, T stage, N stage, and clinical stage were significantly associated with risk score (**Figure 5A**). Scatter plots are presented using the Wilcoxon signed rank test to display the correlation of risk scores with significant clinicopathological traits (**Figures 5B–E**). Next, we found T stage (P < 0.001, HR = 1.550, 95% CI: 1.349-1.782), N stage (P < 0.001, HR = 1.604, 95% CI: 1.446-1.779), M stage (P < 0.001, HR = 2.687, 95% CI: 2.033-3.552), clinical stage (P < 0.001, HR = 1.901, 95% CI: 1.686-2.142), and risk score (P < 0.001, HR = 1.310, 95% CI: 1.216-1.413) showed statistical differences by univariate cox regression analysis (**Figure 5F**), while in the multivariate cox regression analysis, only clinical stage (P < 0.001, HR = 1.845, 95% CI: 1.441-2.363) and riskScore (P < 0.001, HR = 1.212, 95% CI: 1.121-1.310) were considered as independent prognostic factors (**Figure 5G**). The detailed values for univariate and multivariate cox regression analyses are shown in **Table S1**. Combined with the clinicopathological information, ROC analysis showed that the AUC for risk score (0.695) and clinical stage (0.682) was greater than 0.65, which suggested that risk score and clinical stage had certain predictive ability in the prognosis of digestive pan-cancer (**Figure 5H**). Based on a multivariate cox regression analysis, we adopted a nomogram that included clinicopathological characteristics and risk score. As shown in the nomogram, risk score and clinical stage have the greatest impact on 1-, 3-, and 5-year survival in patients with digestive system pan-cancer (**Figure 6**). The C-index of the prognostic model was 0.713 (95% CI: 0.698-0.728). The risk scores increased with clinical stage, indicating that this prognostic model is significantly associated with the progression of pan-cancer of the digestive system (**Table 2**).

**Table 2:** Clinical influences of prognostic model in digestive system pan-cancer data.

Clinical		n	Risk score		t	p
			Mean	SD		
**Age**						
	<65	554	1.127	0.779	2.461	0.014
	≥65	566	1.034	0.432		
**Sex**						
	FEMALE	413	1.003	0.368	-3.653	<0.01
	MALE	707	1.124	0.737		
**T**						
	T1	157	0.945	0.291	-4.557	<0.01
	T2	225	1.193	0.738		
	T3	600	1.055	0.656		
	T4	138	1.155	0.566		
**M**						
	M0	1011	1.073	0.523	-0.576	0.566
	M1	109	1.142	1.242		
**N**						
	N0	636	1.012	0.475	-3.695	<0.01
	N1	264	1.166	0.601		
	N2	155	1.05	0.513		
	N3	65	1.458	1.531		
**Stage**						
	i	242	0.954	0.384	-4.359	<0.01
	II	387	1.125	0.599		
	III	370	1.082	0.457		
	IV	121	1.178	1.245		

### Prediction of Tumor-Infiltrating Immune Cells With Prognostic Model

**Figure 7:**
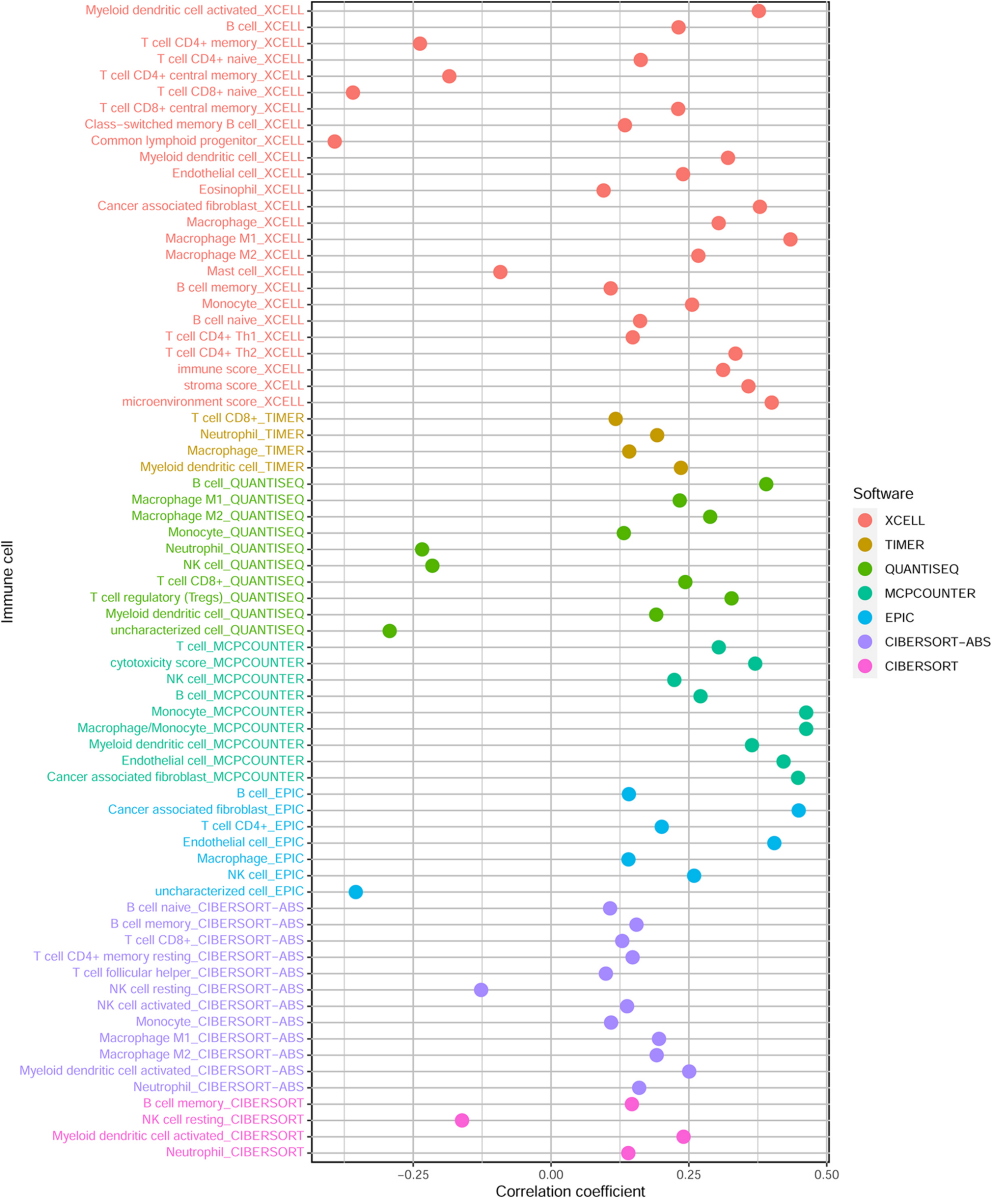
The relationship between risk scores and tumor-infiltrating immune cells.

To investigate whether the HRlncRNAs are associated with the tumor immune microenvironment, we assessed the relationship between the risk score and tumor-infiltrating immune cells by the Wilcoxon signed-rank test. The results indicated that high-risk group was more positively correlated with tumor-infiltrating immune cells such as B cells, cancer associated fibroblast, endothelial cell, monocyte, macrophages, and myeloid dendritic cell, whereas they were negatively correlated with CD4+ T cells (**Figure 7** and **Figure S2**).

### Prediction of Immunosuppressive Molecules With Prognostic Model

**Figure 8:**
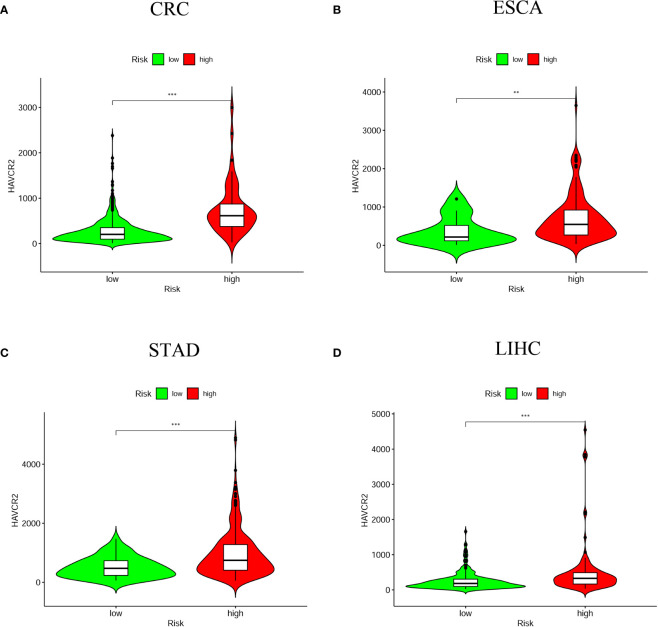
The relationship between risk scores and immune checkpoint inhibitors. HAVCR2 was positively correlated with high-risk groups in the four digestive system tumors **(A)** CRC; **(B)** ESCA; **(C)** STAD; **(D)** LIHC (**P < 0.01; ***P < 0.001).

As ICI has been considered as an effective drug for cancer treatment in recent years, we investigated whether it associated the prognostic model with ICI-related biomarkers. The results showed that high-risk score group was positively correlated with high expression of HAVCR2 in four digestive system cancers (**Figure 8**); with high expression of CD274 in CRC, ESCA and STAD; with high expression of PDCD1 in CRC, ESCA and LIHC; with high expression of LAG3 in CRC and STAD; with high expression of CTLA4 and TIGIT in CRC and LIHC (P < 0.05) (**Figure S3**).

### Analysis of the Correlation Between the Risk Score and Chemotherapeutics

**Figure 9:**
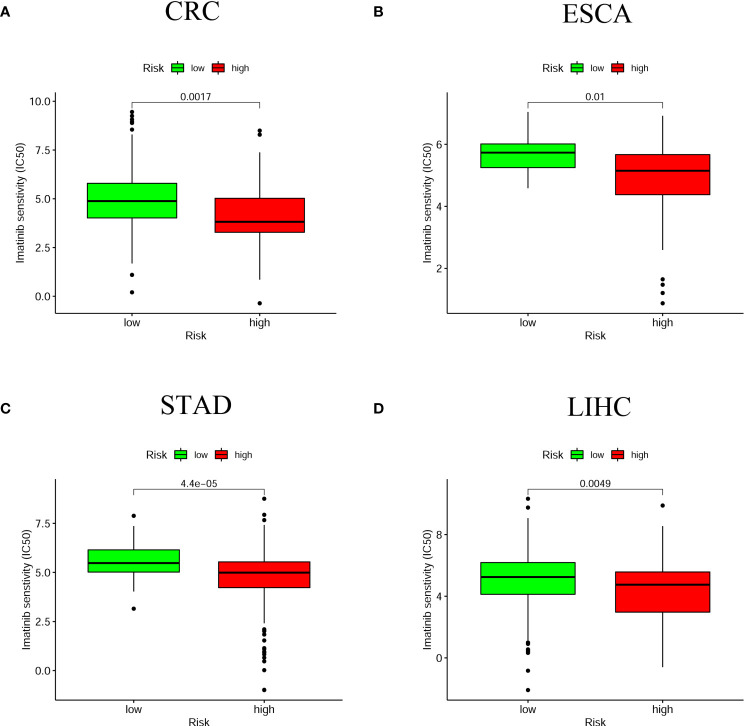
The relationship between risk scores and anti-tumor drugs. The high-risk group in the four digestive system tumors was associated with a lower IC50 for Imatinib **(A)** CRC; **(B)** ESCA; **(C)** STAD; **(D)** LIHC. P < 0.05 was significant.

Besides immune checkpoint inhibitor treatment, we also attempted to identify correlations between risk score and the effectiveness of five common anticancer drugs. We found that high-risk score groups were associated with lower half-inhibitory centration (IC50) of Imatinib anticancer drug among four digestive system cancers (**Figure 9**). Whereas the correlations of IC50 of other anticancer drugs with the high-risk score groups were mainly in CRC and LIHC, such as Gemcitabine, Cisplatin, Doxorubicin, and Mitomycin (**Figure S4**). This suggests that this prognostic model for the assessment of anticancer drugs is more beneficial for patients with CRC and LIHC.

## Discussion

Malignant tumors of the digestive system are the most common malignancies, with the new morbidity and mortality of which are among the leading in China (20). At present, however, most studies are limited to the analysis of pivotal biomarkers for a single cancer. The concept of digestive system pan-cancer was born out of the fact that tumors of similar tissue origin share certain molecular characteristics. Meanwhile, hypoxia is one of the most common causes of carcinogenesis (4). The hypoxic environment is the outcome of an imbalance between the increased oxygen demand and insufficient oxygen supply, which applies to the high proliferation rate of tumors (5). Tumor hypoxia can trigger a variety of biological processes including metabolic changes, angiogenesis and metastasis (21, 22). In many solid cancers, the significant crosstalk between hypoxia and cancer features promotes malignant progression and diminished anti-tumor responses, leading to resistance to therapy and poor clinical outcomes (23). Hypoxia is therefore emerging as a novel biomarker and target for cancer therapy.

In this study, HRlncRNAs were screened by analyzing lncRNAs co-expression with hypoxia-related genes in four digestive system carcinomas. It then intersected lncRNAs from four tumors to get co-expressed HRlncRNAs. Further, we established the prognostic model of six HRlncRNAs (LUCAT1, MIR4435-2HG, LINC01711, AP000695.2, ADAMTS9-AS2, and AC087521.1) by means of univariate and multivariate cox and lasso regression analyses to explore the potential influence in pan-cancer of the digestive system. To validate the effect of the model on digestive system pan-cancer, we calculated the AUC values under the ROC curve to obtain the optimal model and computed the AIC values at each point on the AUC to identify the optimal cut-off points for high- and low-risk groups of patients with digestive system cancers. Afterwards, we assessed this new model for survival outcomes, clinicopathological characteristics, and immune infiltration cells, immune checkpoint-related biomarkers, and anticancer drugs.

Five of the six lncRNAs have been reported in the literature to be significantly associated with the prognosis of digestive malignancies, including LUCAT1 (24–27), ADAMTS9-AS2 (28–31), MIR4435-2HG (32–34), LINC01711 (35), AP000695.2 (36), while there are no relevant articles concerning the biological function of AC087521.1. LUCAT1, located on the antisense strand in the q14.3 region of chromosome 5, was first identified in smoking-associated lung cancer. Xing et al. (37) reported that LUCAT1 serves an essential role in four digestive system tumors, which contributes to tumor proliferation, migration and invasion. ADAMTS9-AS2, a tumor suppressor, was mainly involved in the PI3K/AKT signaling pathway and epithelial mesenchymal transition (EMT). Xu et al. (38) showed that upregulation of ADAMTS9-AS2 expression inhibited the proliferation, migration and invasion of cancer cells and induced apoptosis. Nötzold et al. (39) suggested that MIR4435-2HG functions as a critical player in cell cycle progression through mitosis. MIR4435-2HG as ceRNA regulates YAP1 oncogenic activity in colorectal cancer *via* sponge-adsorbed miR-206 (40). In STAD, MIR4435-2HG is engaged in the development, metastasis and EMT of gastric cancer cells through mediating the miR-138-5p/Sox4 axis (41). In LIHC, MIR4435-2HG promotes cancer cell proliferation through upregulation of miRNA-487a (42). Xu et al. (35) found that LINC01711, which was originated from the exosomes of cancer cells and closely correlated with the intracellular concentration of donor cells, could promote the proliferation, migration and invasion of esophageal cancer cells and inhibit apoptosis by upregulating FSCN1 and downregulating miR-326, thus promoting the development of ESCA. In the construction of a prognostic model, Zha et al. (36) found that AP000695.2, as an oncogenic factor, was positively correlated with the TNM stage and could be an independent prognostic factor for gastric cancer. Therefore, the combined model can be considered as new prognostic biomarkers for further studies.

Recently, the advent of immunotherapy as a potentially effective approach to the treatment of different cancer types, including those of the digestive system, has drawn increasing attention to cancer immunotherapy (43). Tumor hypoxia is an enormous barrier to effective cancer treatment, particularly immunotherapy, because of the irregular growth kinetics of tumors resulting in increased oxygen consumption (9, 44). Hence, there is a great deal of research focused on improving hypoxia in order to revolutionize tumor therapy by blocking immunosuppression or immune escape (45, 46).

In this study, the relationship of risk score and tumor-infiltrating immune cells was predicted by seven algorithms, including XCELL (47, 48), TIMER (49, 50), QuanTIseq (51), MCP-counter (52, 53), EPIC (54), CIBERSORT-ABS (55), and CIBERSORT (56). We found that B cells, cancer associated fibroblast, endothelial cell, monocyte, macrophages, and myeloid dendritic cell were significantly associated with high-risk score. Assuming that hypoxia leads to immunosuppression in the high-risk group, the existence of these immune cells could enhance the efficacy of immunotherapy in patients with digestive system pan-cancer. Previous evidence suggested that tumor-infiltrating B lymphocytes (TIBs) inhibit tumor progression by secreting immunoglobulins, promoting T cell responses and directly killing cancer cells (57). Helmink et al. (58) found that B cell and tertiary lymphoid structures have a potential effect in the response to immune checkpoint blockade (ICB) treatment. The endothelial cells and tumor-associated fibroblasts are the major inducers of the migration and invasive capacity of cancer cells and may be novel targets for cancer therapy (59, 60). Previous studies have shown that TME influences the differentiation of monocytes to macrophages and dendritic cells (61). Similarly, hypoxia, and in particular HIF-1α, drives the differentiation of myeloid-derived suppressor cells (MDSCs) into tumor-associated macrophages (TAMs), which guides cancer treatment. For instance, the restriction of TAMs to normoxic regions by blocking the Sema3A/Neuropilin-1 pathway reduced tumor pro-angiogenic and immunosuppressive functions and inhibited tumor development and metastasis (62). Thus, the positive correlation of these immune cells with high-risk group proved that the prognostic model has potential for immunotherapy. We then examined that relationship between common immune checkpoint inhibitors and chemotherapy and the prognostic model, which revealing that HAVCR2, the immune checkpoint marker gene, was positively associated with the high-risk group for all four digestive cancers, while other marker genes (CD274, PDCD1, CTLA4, LAG3 and TIGIT) were more significant in some single cancers such as CRC, STAD and LIHC. The lower IC50 for Imatinib was more sensitive to the high-risk group for all four digestive cancers, while several other anti-tumor drugs (Doxorubicin, Mitomycin, Gemcitabine and Cisplatin) were significant in some individual cancers such as CRC and LIHC. Generally, immune-targeted therapies support the production of more new antigens and inhibit the progression of malignant tumors by targeted destruction of cancer cells. As a result, immunotherapies offer more benefit than conventional anticancer treatment. While targeted immunotherapies such as anti-PD1/PDL1 have yielded substantial clinical outcomes for cancer treatment (63), we believe that the identification and validation of specific biomarkers may be of greater benefit to clinical treatment due to the differences between different immune cells and immune checkpoint marker genes.

However, there are a few shortcomings to this study. To begin with, we only downloaded transcriptomic data from the TCGA database for the analysis of digestive system pan-cancer, and the raw data were relatively limited. Moreover, we should perform functional validation of the HRlncRNAs for constructing prognostic model to further ensure the robustness of HRlncRNAs in cancers of the digestive system. In future researches, we hope to supplement the above limitations and ultimately make the study of digestive system pan-cancer more conducive to clinical treatment.

In conclusion, our research suggested that the prognostic model constructed by HRlncRNAs and validated at multiple levels could predict the prognosis of digestive system pan-cancer and might help patients to benefit from anti-tumor immunotherapy.

## Data Availability Statement

The datasets presented in this study can be found in online repositories. The names of the repository/repositories and accession number(s) can be found below: The Cancer Genome Altas database.

## Author Contributions

RC and ZH contributed to the design of the study and the writing of the manuscript. ZH, HL, and HG conducted the writing of the manuscript. ZH, JJ, and YJ performed the bioinformatics analysis. NZ, ZS, XW, PS, and HW reviewed and revised the manuscript. All authors contributed to the article and approved the submitted version.

## Funding

This study was supported by 2019 Scientific Research Project of Basic Scientific Research Business Fee of Provincial Higher Education Institutions of Heilongjiang Province (NO.2019-KYYWFMY-0001) and the PhD Start-up Fund of Mudanjiang Medical University (NO.2021-MYBSKY-041).

## Conflict of Interest

The authors declare that the research was conducted in the absence of any commercial or financial relationships that could be construed as a potential conflict of interest.

## Publisher’s Note

All claims expressed in this article are solely those of the authors and do not necessarily represent those of their affiliated organizations, or those of the publisher, the editors and the reviewers. Any product that may be evaluated in this article, or claim that may be made by its manufacturer, is not guaranteed or endorsed by the publisher.
