# Supplementary material for: Construction of a Prognostic Model for Hypoxia-Related LncRNAs and Prediction of the Immune Landscape in the Digestive System Pan-Cancer
Source: Front Oncol. 2022 Apr 27;12:812786. doi: 10.3389/fonc.2022.812786 (PMC9092832; doi:10.3389/fonc.2022.812786)
Supplement: Supplementary file 1 [file Image_1.pdf]

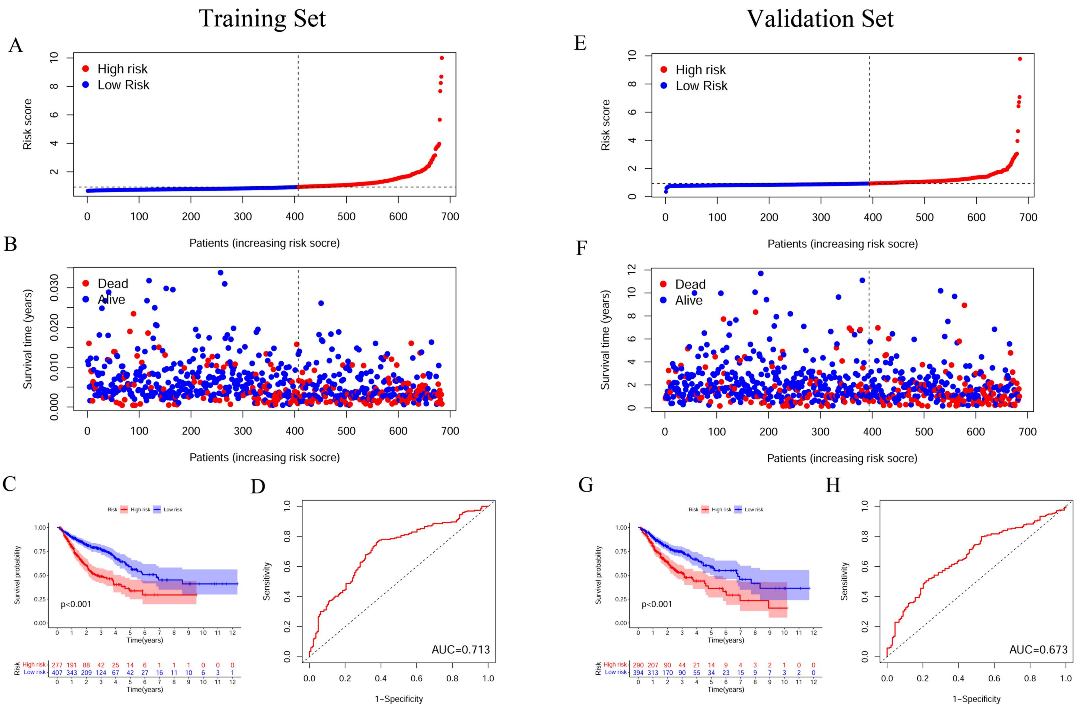

Supplement Figure 1. The assessment of prognostic model in training set (A-D) and validation set (E-H). (A, E) As the number of patients increased, the risk scores of the prognostic model were raised. (B, F) As the number of patients increased, the number of patient deaths significantly increased. (C, G) The survival time was significantly longer in the low-risk group than in the high-risk group. (D, H) ROC analysis based on three years survival outcomes showed that the prognostic models had certain predictive ability.
