# Supplementary material for: Construction of a Prognostic Model for Hypoxia-Related LncRNAs and Prediction of the Immune Landscape in the Digestive System Pan-Cancer
Source: Front Oncol. 2022 Apr 27;12:812786. doi: 10.3389/fonc.2022.812786 (PMC9092832; doi:10.3389/fonc.2022.812786)
Supplement: Supplementary file 2 [file Image_2.pdf]

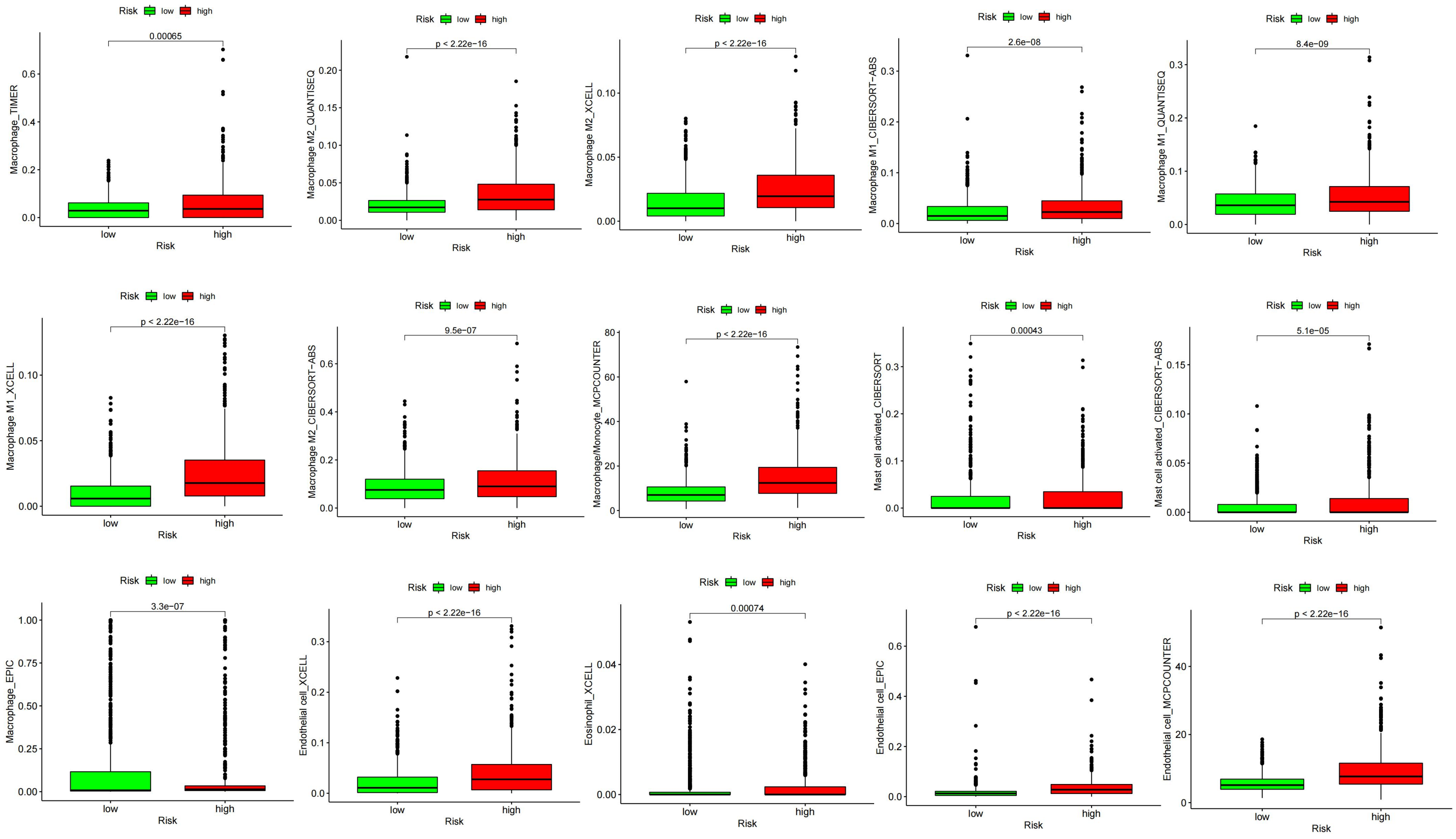

Risk low high
0.00065
Macrophage_TIMER
p < 2.22e−16
Macrophage M2_QUANTISEQ
p < 2.22e−16
Macrophage M2_XCELL
2.6e−08
Macrophage M1_CIBERSORT−ABS
8.4e−09
Macrophage M1_QUANTISEQ
p < 2.22e−16
Macrophage M1_XCELL
9.5e−07
Macrophage M2_CIBERSORT−ABS
p < 2.22e−16
Macrophage/Monocyte_MCPCOUNTER
0.00043
Mast cell activated_CIBERSORT
5.1e−05
Mast cell activated_CIBERSORT−ABS
3.3e−07
Macrophage_EPIC
p < 2.22e−16
Endothelial cell_XCELL
0.00074
Eosinophil_XCELL
p < 2.22e−16
Endothelial cell_EPIC
p < 2.22e−16
Endothelial cell_MCPCOUNTER
low high
Risk

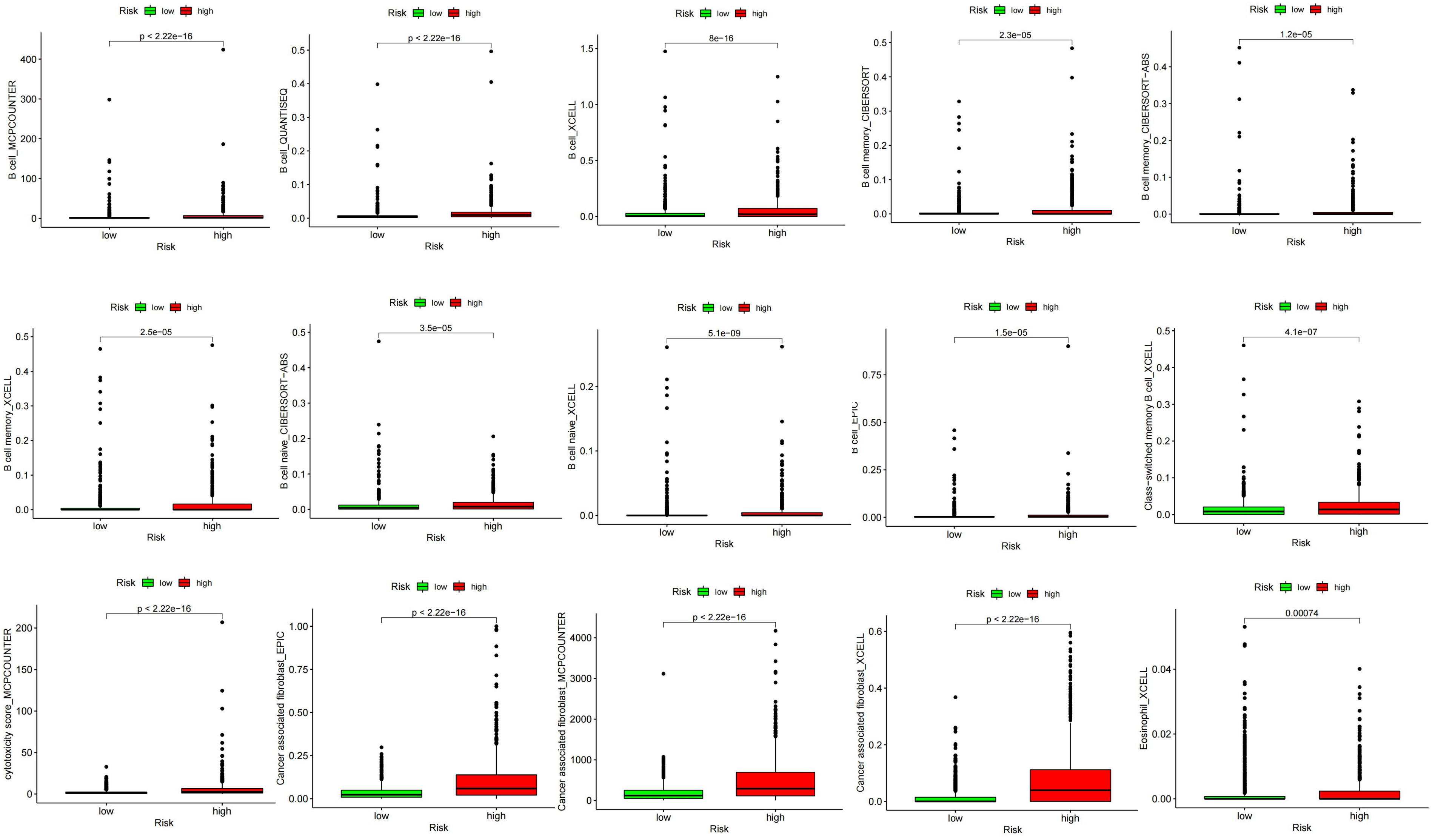
Risk low high
B cell_MCPCOUNTER
p < 2.22e-16
B cell_QUANTISEQ
p < 2.22e-16
B cell_XCELL
8e-16
B cell memory_CIBERSORT
2.3e-05
B cell memory_CIBERSORT-ABS
1.2e-05
B cell memory_XCELL
2.5e-05
B cell naive_CIBERSORT-ABS
3.5e-05
B cell naive_XCELL
5.1e-09
1.5e-05
Class-switched memory B cell_XCELL
4.1e-07
cytotoxicity score_MCPCOUNTER
p < 2.22e-16
Cancer associated fibroblast_EPIC
p < 2.22e-16
Cancer associated fibroblast_MCPCOUNTER
p < 2.22e-16
Cancer associated fibroblast_XCELL
p < 2.22e-16
Eosinophil_XCELL
0.00074
low
high
Risk

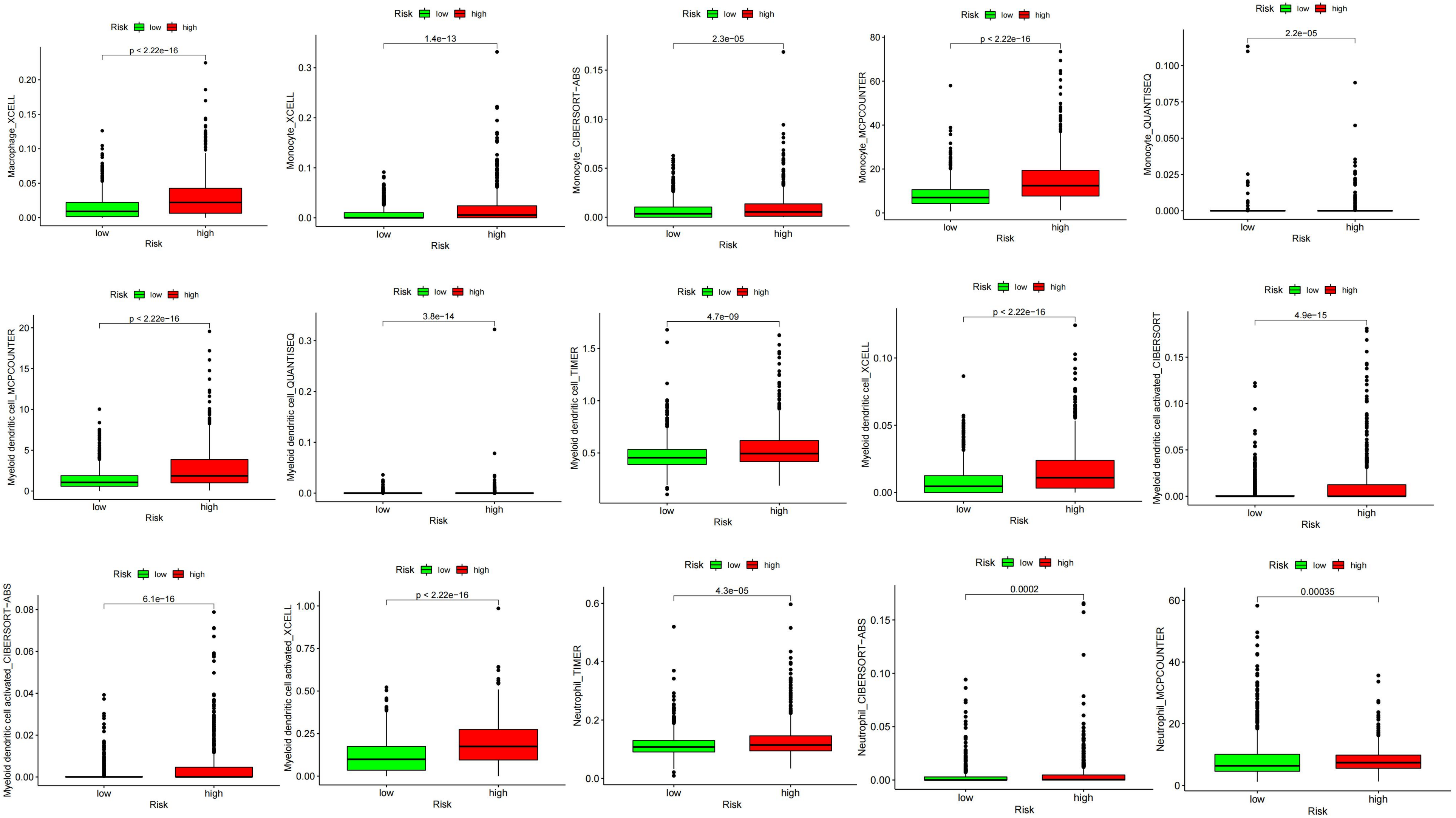
Risk low high
p < 2.22e−16
Macrophage_XCELL
Risk
1.4e−13
Monocyte_XCELL
2.3e−05
Monocyte_CIBERSORT−ABS
Monocyte_MCPCOUNTER
2.2e−05
Monocyte_QUANTISEQ
Myeloid dendritic cell_MCPCOUNTER
3.8e−14
Myeloid dendritic cell_QUANTISEQ
4.7e−09
Myeloid dendritic cell_TIMER
Myeloid dendritic cell_XCELL
4.9e−15
Myeloid dendritic cell activated_CIBERSORT
6.1e−16
Myeloid dendritic cell activated_CIBERSORT−ABS
Myeloid dendritic cell activated_XCELL
4.3e−05
Neutrophil_TIMER
0.0002
Neutrophil_CIBERSORT−ABS
0.00035
Neutrophil_MCPCOUNTER
low
high

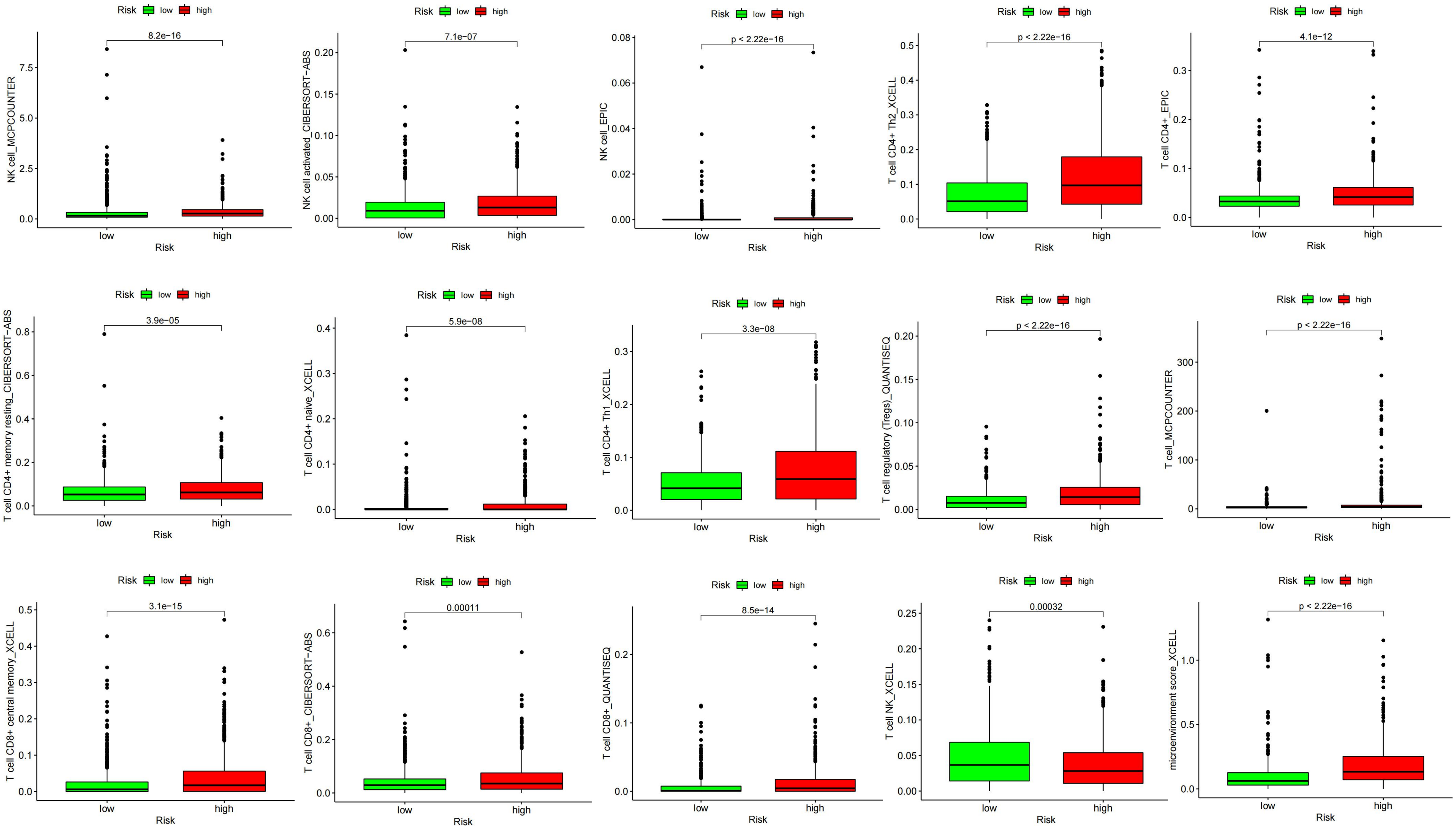

Risk low high
8.2e−16
NK cell_MCPCOUNTER
7.1e−07
NK cell activated_CIBERSORT−ABS
p < 2.22e−16
NK cell_EPIC
p < 2.22e−16
T cell CD4+ Th2_XCELL
4.1e−12
T cell CD4+_EPIC
3.9e−05
T cell CD4+ memory resting_CIBERSORT−ABS
5.9e−08
T cell CD4+ naive_XCELL
3.3e−08
T cell CD4+ Th1_XCELL
p < 2.22e−16
T cell regulatory (Tregs)_QUANTISEQ
p < 2.22e−16
T cell_MCPCOUNTER
3.1e−15
T cell CD8+ central memory_XCELL
0.00011
T cell CD8+_CIBERSORT−ABS
8.5e−14
T cell CD8+_QUANTISEQ
0.00032
T cell NK_XCELL
p < 2.22e−16
microenvironment score_XCELL
low
high
Risk

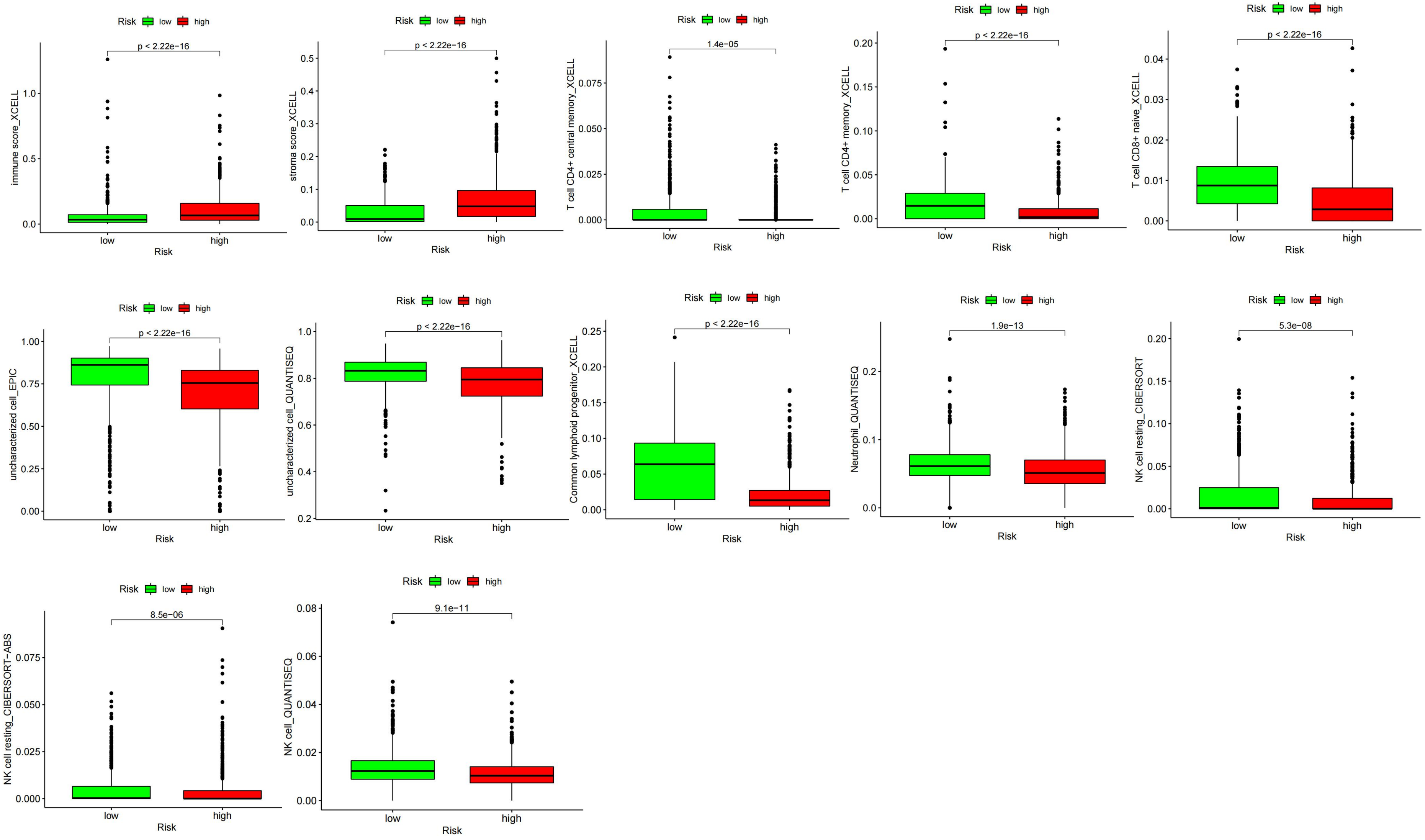

Supplement Figure 2. The box plots showed that relationship between the risk scores and tumor-infiltrating immune cells.
