# Supplementary material for: Construction of a Prognostic Model for Hypoxia-Related LncRNAs and Prediction of the Immune Landscape in the Digestive System Pan-Cancer
Source: Front Oncol. 2022 Apr 27;12:812786. doi: 10.3389/fonc.2022.812786 (PMC9092832; doi:10.3389/fonc.2022.812786)
Supplement: Supplementary file 3 [file Image_3.pdf]

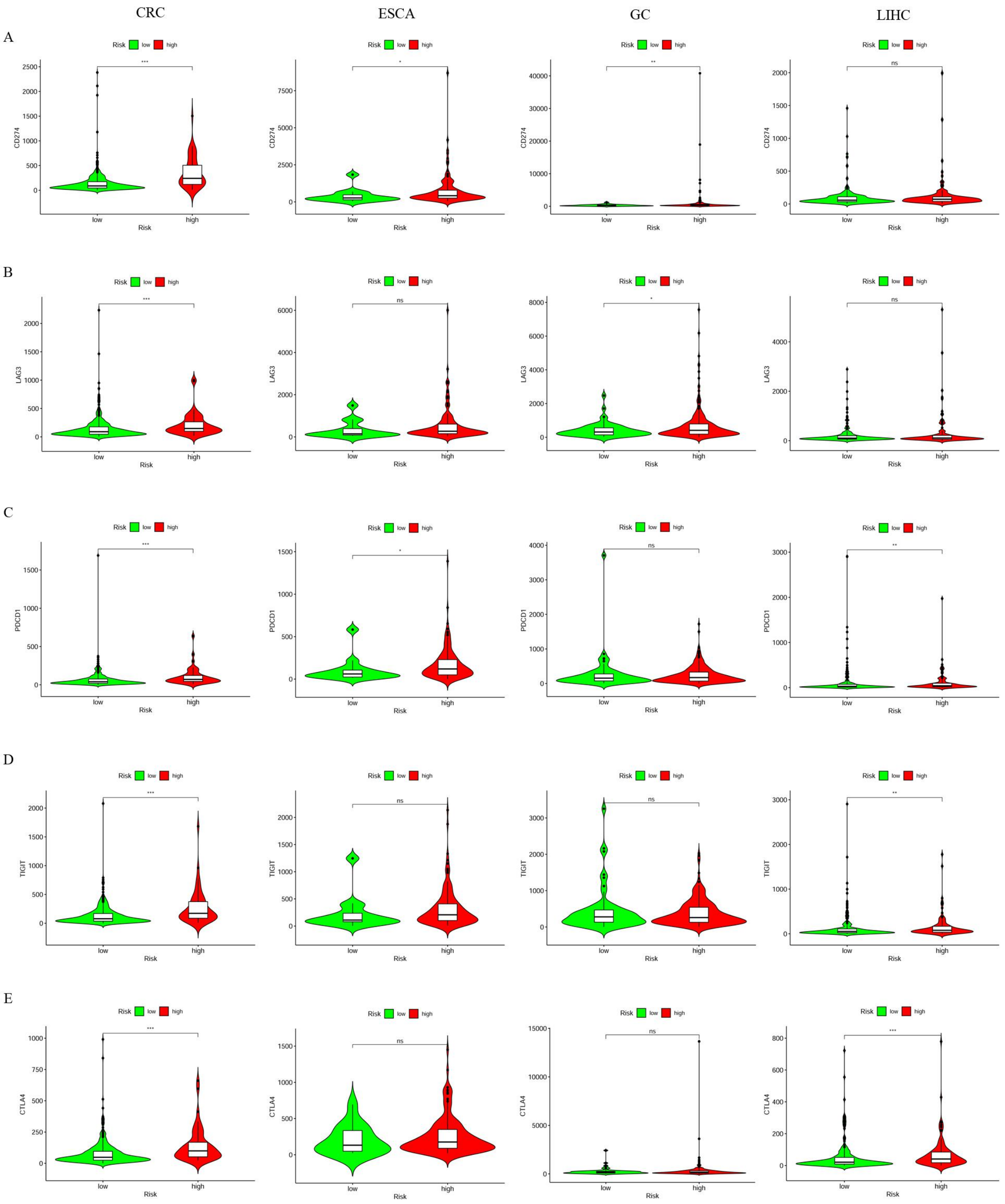

Supplement Figure 3. The relationship between risk scores and immune checkpoint. (A) CD274 was positively associated with high-risk groups among CRC, ESCA, GC; (B) LAG3 in CRC and GC was positively correlated with high-risk groups; (C) PDCD1 for CRC, ESCA and LIHC, (D) TIGIT and (E) CTLA4 among CRC and LIHC were positively associated with high-risk groups. (* $P < 0.05$; ** $P < 0.01$; *** $P < 0.001$)
