# Supplementary material for: Construction of a Prognostic Model for Hypoxia-Related LncRNAs and Prediction of the Immune Landscape in the Digestive System Pan-Cancer
Source: Front Oncol. 2022 Apr 27;12:812786. doi: 10.3389/fonc.2022.812786 (PMC9092832; doi:10.3389/fonc.2022.812786)
Supplement: Supplementary file 4 [file Image_4.pdf]

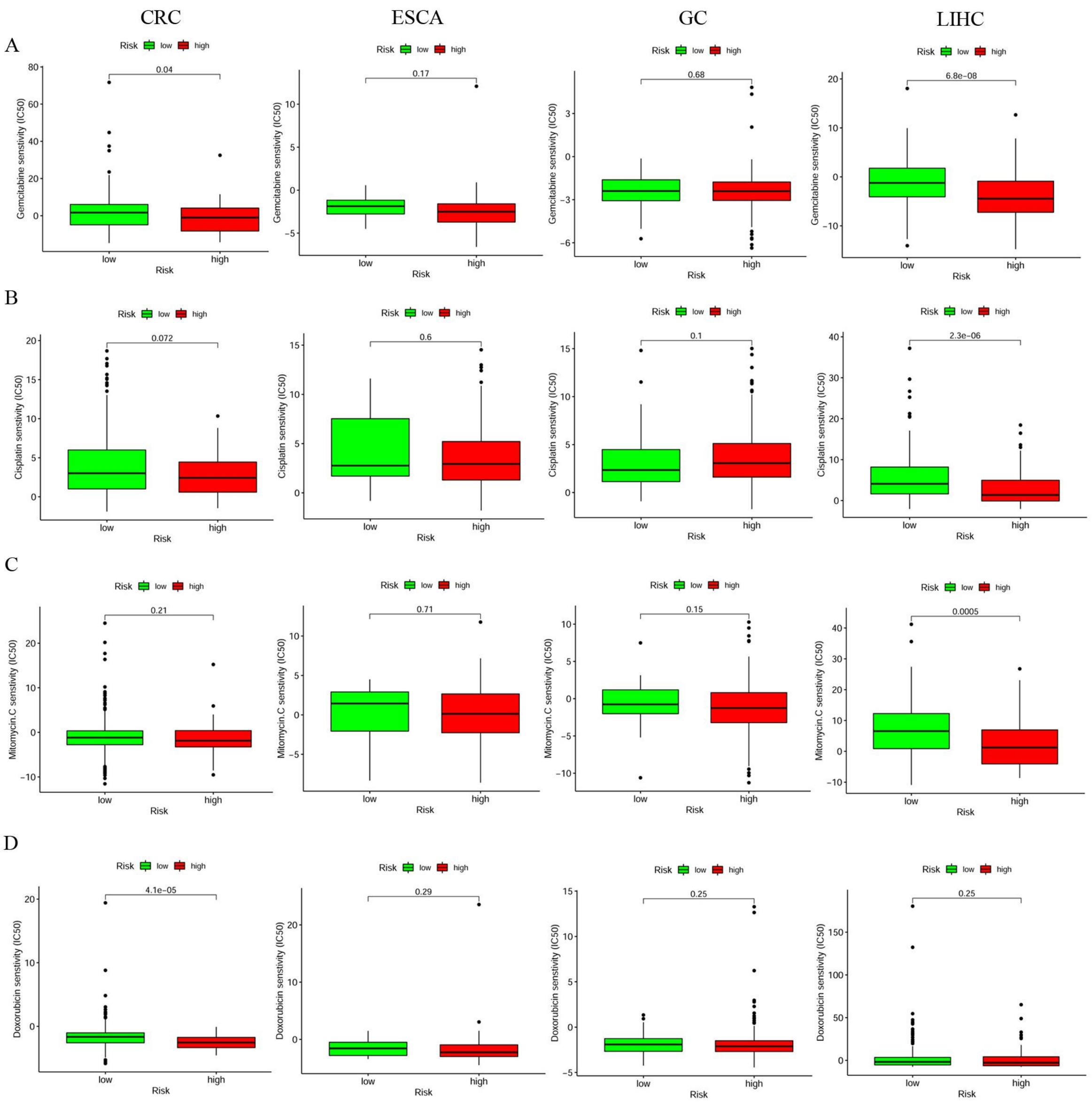


Supplement Figure 4: The relationship between risk scores and common anti-tumor drugs. The high-risk group among CRC and LIHC was associated with a lower IC50 for (A) Gemcitabine; The high-risk group of LIHC was correlated with lower IC50 for (B) Cisplatin and (C) Mitomycin; The high-risk group of CRC correlated with lower IC50 for (D) Doxorubicin. $P < 0.05$ was significant.
