# Supplementary material for: Construction of a Prognostic Model for Hypoxia-Related LncRNAs and Prediction of the Immune Landscape in the Digestive System Pan-Cancer
Source: Front Oncol. 2022 Apr 27;12:812786. doi: 10.3389/fonc.2022.812786 (PMC9092832; doi:10.3389/fonc.2022.812786)
Supplement: Supplementary file 5 [file Table_1.docx]

| **uniCox** |  |  |  |  |
| --- | --- | --- | --- | --- |
|  | HR | HR.95L | HR.95H | pvalue |
| Age | 1.411 | 1.143 | 1.741 | <0.01 |
| Sex | 1.218 | 0.977 | 1.518 | 0.080 |
| T | 1.695 | 1.333 | 2.156 | <0.01 |
| N | 2.222 | 1.770 | 2.790 | <0.01 |
| M | 2.622 | 2.002 | 3.436 | <0.01 |
| Stage | 2.645 | 2.131 | 3.284 | <0.01 |
| riskScore | 1.490 | 1.329 | 1.669 | <0.01 |
| **multiCox** |  |  |  |  |
|  | HR | HR.95L | HR.95H | pvalue |
| Age | 1.562 | 1.262 | 1.934 | <0.01 |
| T | 0.968 | 0.726 | 1.292 | 0.827 |
| N | 1.249 | 0.964 | 1.620 | 0.093 |
| M | 1.665 | 1.243 | 2.229 | <0.01 |
| Stage | 2.197 | 1.635 | 2.952 | <0.01 |
| riskScore | 1.496 | 1.329 | 1.684 | <0.01 |

**Supplement Table 1: The risk score were significantly associated with clinicopathological characteristics in digestive system pan-cancer by the univariate and multivariate cox analysis.**
